# Supplementary material for: Combined MEK and JAK/STAT3 pathway inhibition effectively decreases SHH medulloblastoma tumor progression
Source: Commun Biol. 2022 Jul 14;5:697. doi: 10.1038/s42003-022-03654-9 (PMC9283517; doi:10.1038/s42003-022-03654-9)
Supplement: Supplementary file 2 — Supplementary Information [file 42003_2022_3654_MOESM2_ESM.pdf]

Combined MEK and JAK/STAT3 pathway inhibition effectively decreases SHH medulloblastoma tumor progression

Supplementary Figure and Table Legends

Supplementary Figure 1: The majority of genes are upregulated following MEK inhibition, including those in the JAK/STAT3 pathway.

a. Hierarchical clustering heat map of tumors from vehicle control and selumetinib treated xenografts. Red and blue color represent increased and decreased gene expression, respectively.

b. Reduction of pERK1/2 and pSTAT3 levels with concomitant elevation of cleaved caspase 3 levels in selumetinib treated primary Daoy tumorspheres at day 3. Total ERK, total STAT3 and GAPDH serve as loading controls.

c. Representative images of Daoy (upper) and UI226 (lower) tumorspheres following 1 passage (6 days) in either DMSO or 1 μM selumetinib. Scale bar: 300 μm.

d. Elevated pSTAT3 levels following selumetinib treatment of Daoy tumorspheres over 3 passages. Total STAT3 and GAPDH serve as loading controls.

Supplementary Figure 2: Hierarchical clustering based on entire JAK/STAT signature gene set.

Unsupervised hierarchical clustering restricted to the entire JAK/STAT KEGG gene set across the 4 MB subgroups from the Cavalli et al. dataset representing 763 patient

samples (WNT: N=70 samples in blue; SHH: N=223 samples in red; Group 3: N=144 samples in yellow; Group 4: N=326 samples in green).

Supplementary Figure 3: JAK/STAT3 pathway inhibitors decrease tumorsphere number and size but have no significant effect on cell viability.

a-c. Western blot for pSTAT3 (Tyr705) activation, total STAT3 and GAPDH following treatment of UI226 (a) Daoy (b) or *Ptch*+/-:*p53*+/- (c) SHH MB tumorspheres with AZD1480 for 3 hours.

d. UI226 tumorsphere size following treatment with increasing doses of vincristine. Error bars: SEM. N=3 biological replicates and n=3 technical replicates for each biological replicate. Results were analyzed using one-way ANOVA and a Dunnett's test for multiple comparisons. For 0.3125 μM, p=0.0023 and for all other significant concentrations, p<0.0001.

e-f. Cumulative frequency distribution of tumorsphere size for UI226 (e) and Daoy (f) following treatment with various concentrations of AZD1480. Tumorsphere size was analyzed using 2-sample Kolmogorov–Smirnov tests. p<0.01**, p<0.001***. For UI226 AZD1480 (e): 0.5 μM, p=0.0053; 2.0 μM, p=0.0006. For Daoy AZD1480 (f): 2.0 μM, p<0.0001.

g-l. Total number of UI226 (g-h), Daoy (i-j) and *Ptch*+/-:*p53*+/- SHH MB (k-l) tumorspheres following treatment with pacritinib (g,i,k) and AZD1480 (h,j,l). Error bars: SEM. N=5-9 biological replicates for each experiment. Results were analyzed using one-

way ANOVA and a Dunnett's test for multiple comparisons. For UI226 MB pacritinib treated cells (g): 0.05 μM, p=0.05; 0.1 μM, p=0.0126; 0.25 μM, p=0.0057. For Daoy MB pacritinib treated cells (i): 0.25 μM, p=0.0224. For Daoy MB AZD1480 treated cells (j): 2 μM, p=0.0149. For Mouse SHH MB pacritinib treated cells (k): 0.25 μM, p=0.0383. For Mouse SHH MB AZD1480 treated cells (l): 0.50 μM, p=0.0212; 1.0 μM, p=0.0250; 2.0 μM, p=0.0017.

m-r. Viability of UI226 (m-n), Daoy (o-p) and *Ptch*+/-:*p53*+/- SHH MB (q-r) primary tumorspheres as measured by Trypan blue staining following treatment with pacritinib (m,o,q) and AZD1480 (n,p,r). Error bars: SEM. Results were analyzed using one-way ANOVA and a Dunnett's test for multiple comparisons.

Supplementary Figure 4: MEK + JAK/STAT3 inhibition significantly reduces cell viability in UI226 tumorspheres only.

a-c. Quantification of Trypan Blue staining in UI226 (a), Daoy (b) and *Ptch*+/-:*p53*+/- SHH MB (c) tumorspheres to assess the effects on viability following pacritinib + selumetinib or AZD1480 + selumetinib treatment. Error bars: SEM. N=3-4 biological replicates for Trypan Blue staining. Results were analyzed using ANOVA followed by a Tukey's test for multiple comparisons. For UI226 treated cells (a): DMSO vs pac/sel, p=0.0079; DMSO vs AZD/sel, p<0.0001; AZD vs AZD/sel, p=0.0004. For Daoy treated cells (b): DMSO vs pac/sel, p=0.0288

d-e. Quantification of dying (Annexin V+/7AAD-) and dead (Annexin V+/7AAD+) cells in UI226 tumorspheres following treatment with either a combination of pacritinib + selumetinib (d) or AZD1480 + selumetinib (e). Error bars: SEM. Results were analyzed using ANOVA followed by a Tukey's test for multiple comparisons. For combinations with pacritinib (d): DMSO vs pac/sel, p=0.0019; pac vs pac/sel, p=0.0283; sel vs pac/sel, p=0.0489. For combinations with AZD1480 (e): DMSO vs AZD/sel, p=0.0314; AZD vs AZD/sel, p=0.0377.

Supplementary Figure 5: Digital spatial profiling reveals no significant differences between protein levels in the tumor core relative to the border regions following UI226 medulloblastoma treatment with pacritinib and/or selumetinib.

a-d. Volcano plots displaying differentially expressed proteins between the tumor core vs. border ROIs for the vehicle (a), pacritinib (b), selumetinib (c) and pacritinib + selumetinib-treated (d) tumor samples. FDR: false discovery rate.

e. Representative immunofluorescent image depicting ROIs from a HDMB03 Group 3 MB tumor-bearing NOD SCID mouse treated with the dual mTORC1/mTORC2 inhibitor PQR620. Regions were selected to compare protein levels inside the tumor core (blue) and along the tumor border (orange). Sample was stained for MAP2 (green), Ki67 (pink) and Syto13 (blue) for tumor visualization.

f-h. Boxplots depicting select differentially expressed proteins based on signal-to-noise-ratio in vehicle control, and HDMB03 MB tumor-bearing NOD SCID mice treated with either 50 mg/kg or 100 mg/kg PQR620, a dual mTORC1/mTORC2 inhibitor. Significant

differences were observed between regions and treatment. Bars represent minimum and maximum counts. Significance was determined using ANOVA followed by a Tukey's test for multiple comparisons. For MBP (f): vehicle border vs 100 mg/kg border, p=0.0357. For GFAP (g): vehicle inside vs vehicle border, p=0.0070; 50 mg/kg inside vs 50 mg/kg border, p=0.0021; 100 mg/kg inside vs 100 mg/kg border, p<0.0001; vehicle border vs 100 mg/kg border, p=0.0270. For Ki67 (h): vehicle inside vs 100 mg/kg inside, p<0.0001; vehicle inside vs 50 mg/kg inside, p<0.0001. Samples were originally characterized by IHC in Zagozewski et al., Nature Communications, 2020.

Supplementary Figure 6: Digital spatial profiling reveals no significant differences between protein levels in CD271^{high} vs. CD271^{low} regions.

Volcano plot showing that there are no significantly different proteins between CD271^{low} and CD271^{high} ROIs in the pacritinib-treated sample.

Supplementary Figure 7: Original uncropped immunoblots from main and supplementary figures.

a. Unprocessed immunoblots associated with Figure 3 and Supplementary Figure 1.

b. Unprocessed immunoblots associated with Supplementary Figure 3.

Supplementary Table 1: List of antibodies used in this study

Zagozewski et al., Figure S1

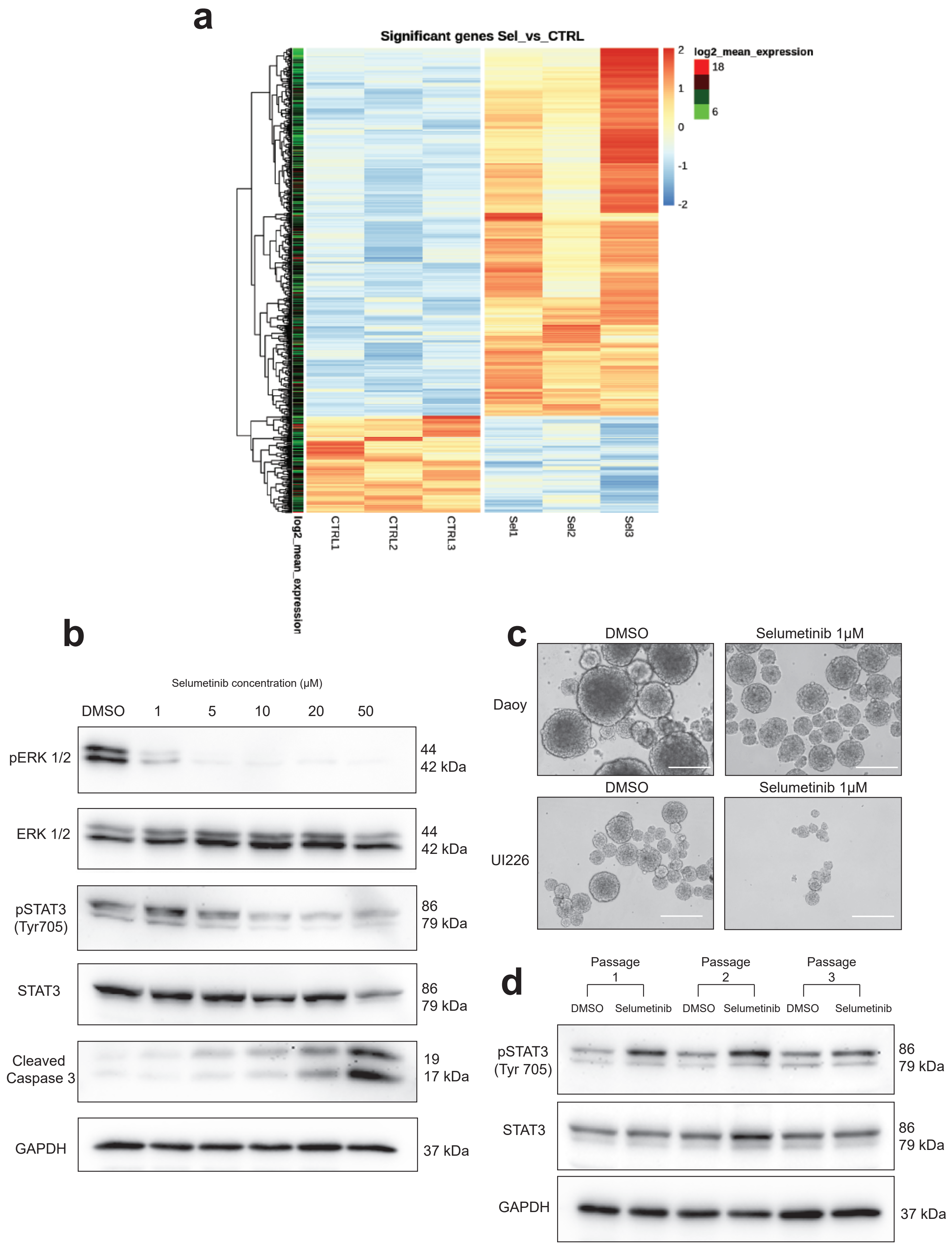

Zagozewski et al., Figure S2

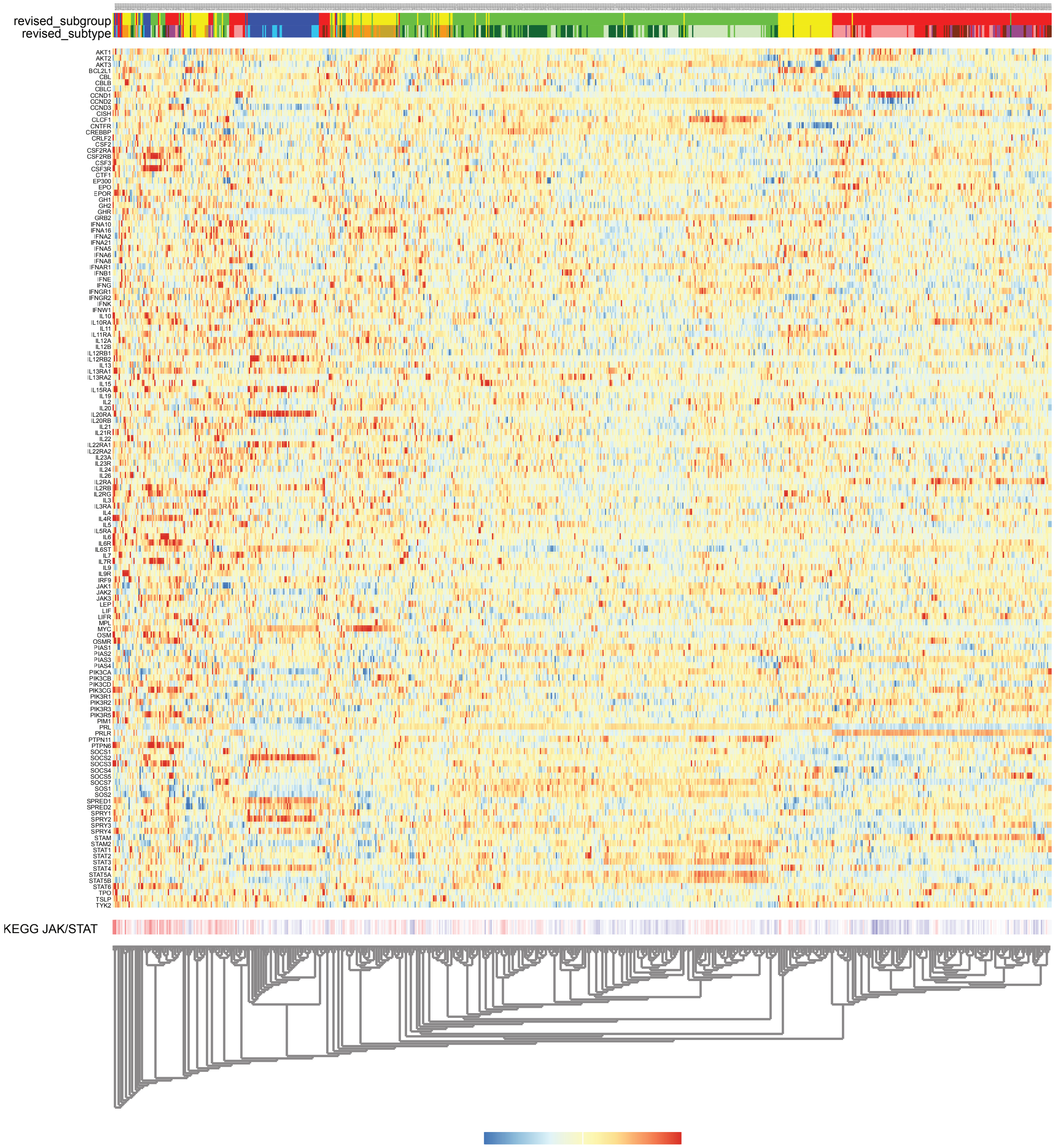

Zagozewski et al., Figure S3

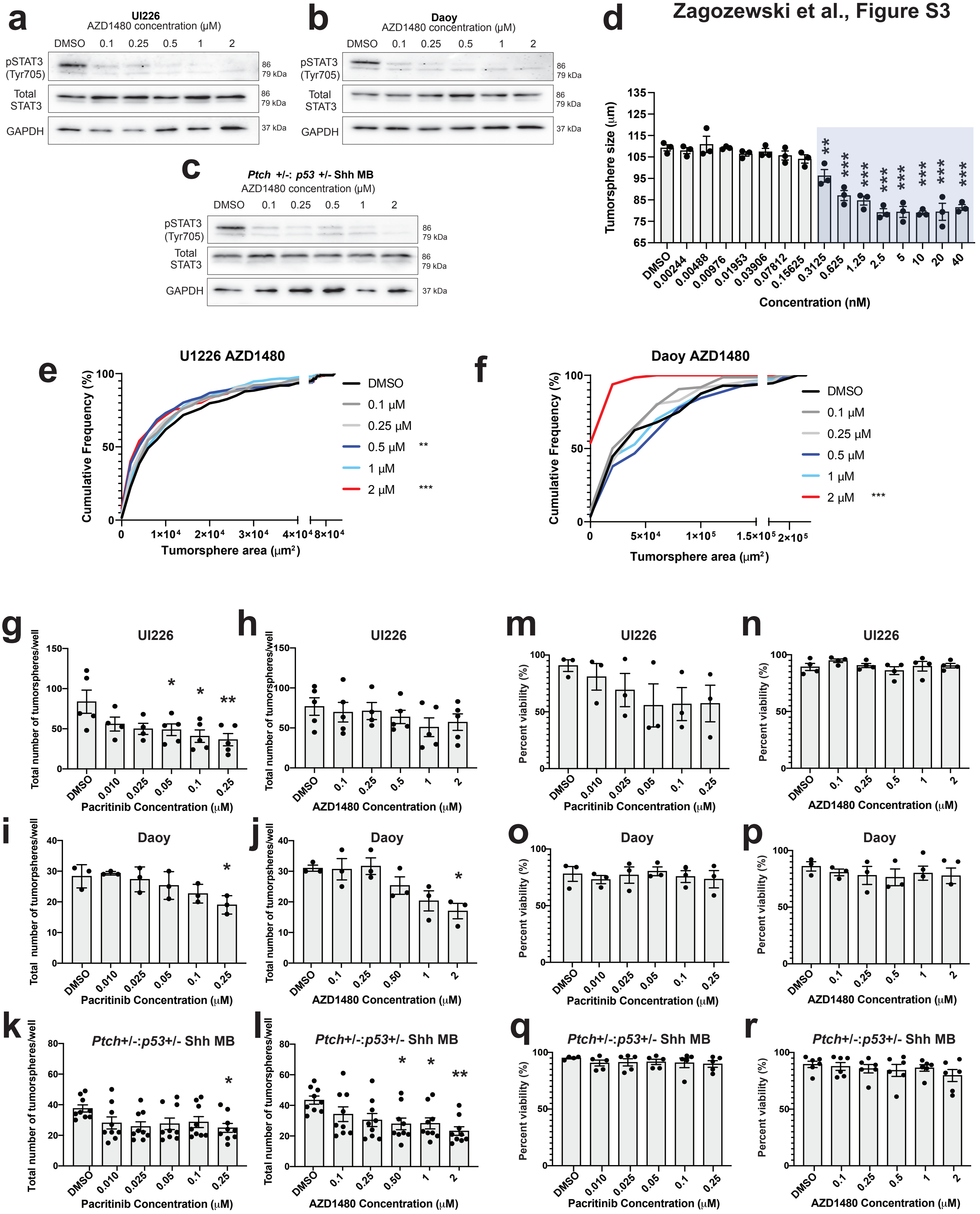

Zagozewski et al., Figure S4

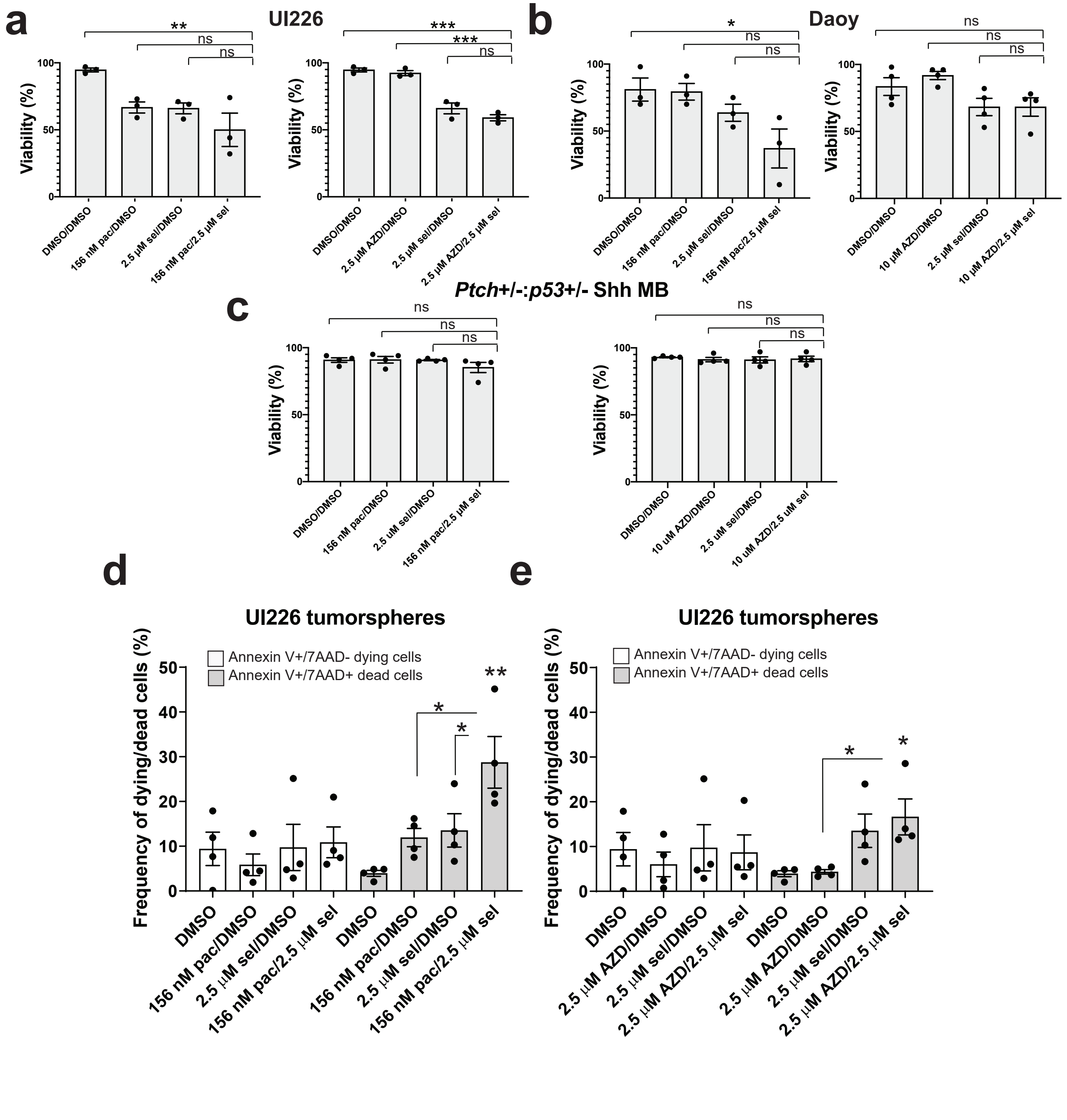

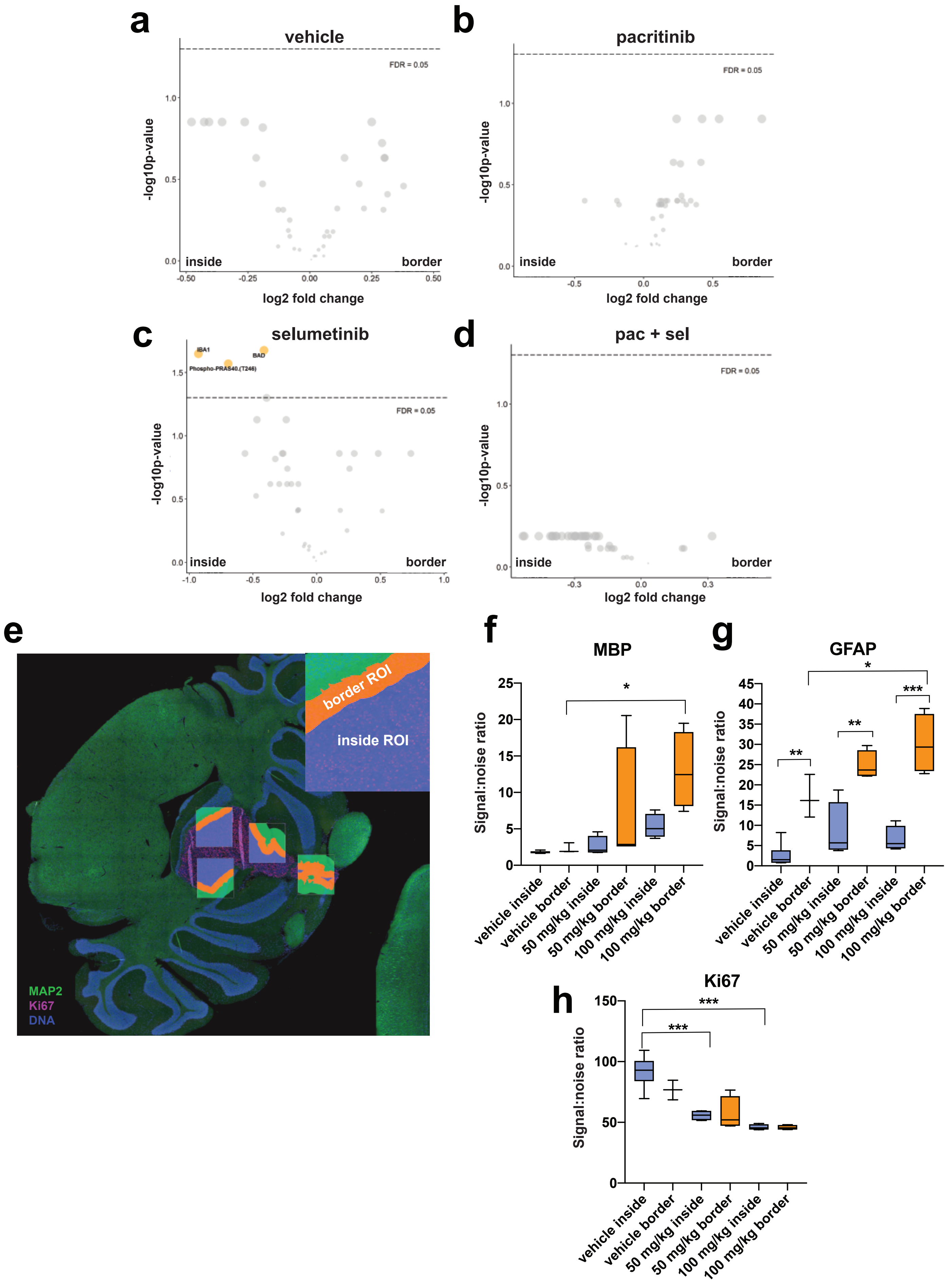
a
vehicle
FDR = 0.05
-log10p-value
inside
border
log2 fold change
b
pacritinib
FDR = 0.05
-log10p-value
inside
border
log2 fold change
c
selumetinib
IBA1
BAD
Phospho-PRAS40.(T246)
FDR = 0.05
-log10p-value
inside
border
log2 fold change
d
pac + sel
FDR = 0.05
-log10p-value
inside
border
log2 fold change
e
border ROI
inside ROI
MAP2
Ki67
DNA
f
MBP
Signal:noise ratio
vehicle inside
vehicle border
50 mg/kg inside
50 mg/kg border
100 mg/kg inside
100 mg/kg border
g
GFAP
Signal:noise ratio
h
Ki67
Signal:noise ratio

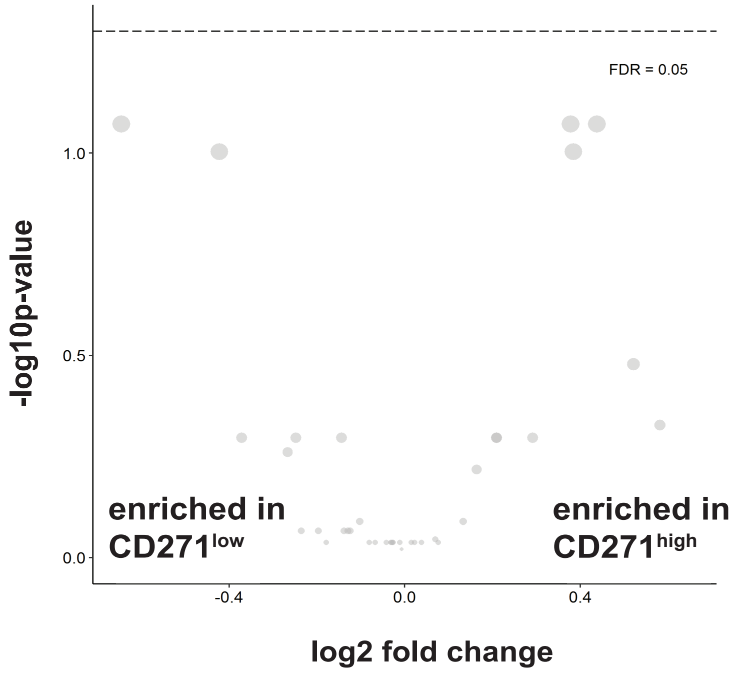
FDR = 0.05
1.0
0.5
0.0
-log10p-value
enriched in
CD271low
enriched in
CD271high
-0.4
0.0
0.4
log2 fold change

Zagozewski et al., Figure S7a

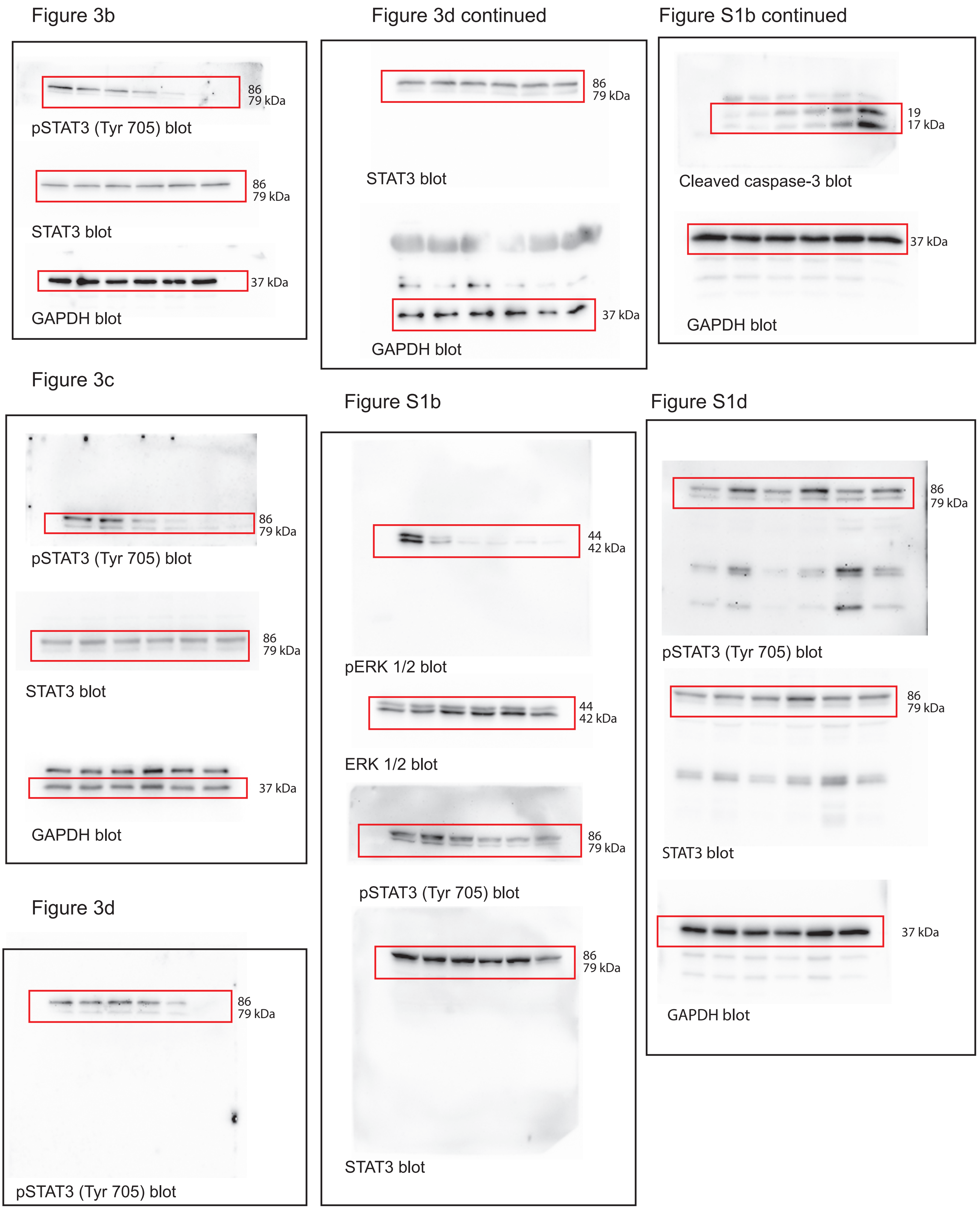

Figure S3a

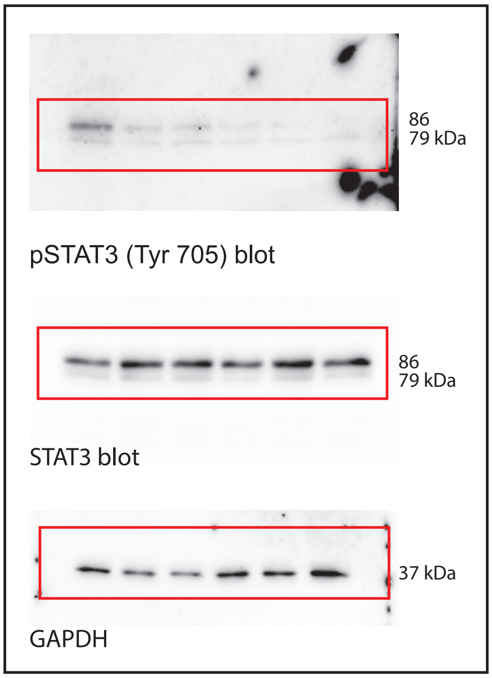

Figure S3b

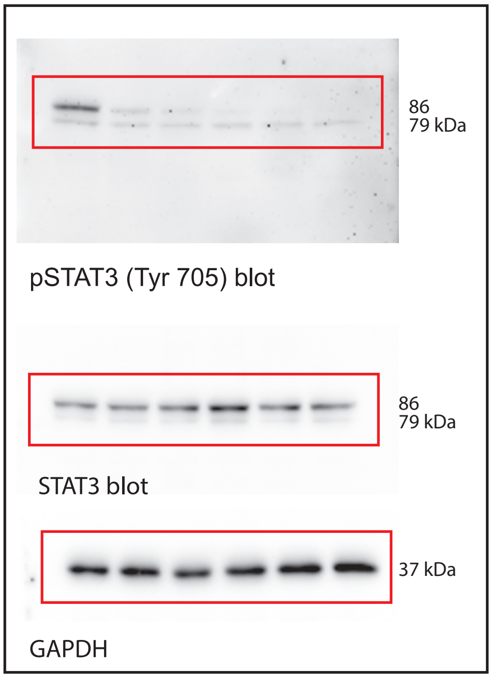

Figure S3c

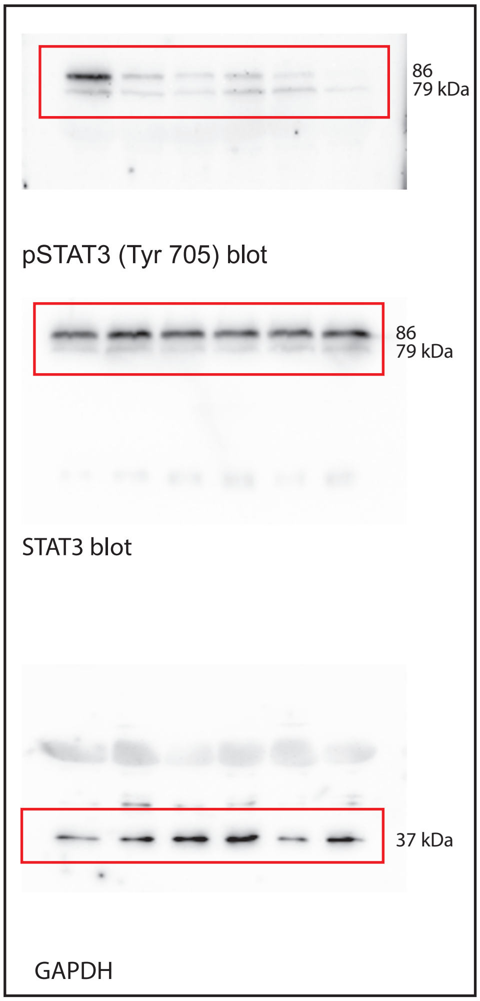

Supplementary Table 1: List of antibodies used in the study

**Immunoblotting**		
Name	Company/Catalogue number	Dilution
Cleaved Caspase-3	CST (9664)	1/1000
GAPDH	SCBT (sc-47724)	1/1000
ERK	CST (4695)	1/1000
pERK	CST (4370)	1/1000
STAT3	CST (4904S)	1/500
p-STAT3	CST (4113S)	1/500
**Immunoblotting secondary antibodies**		
Goat anti-mouse HRP	Abcam (ab6789)	1/3000
Donkey anti-rabbit HRP	Jackson ImmunoResearch (711-035-152)	1/5000
**Immunohistochemistry**		
STEM121	Clontech (Y40410)	1/500
**Immunohistochemistry secondary antibodies**		
Biotin-SP sheep anti-mouse IgG	Jackson ImmunoResearch (515-065-003)	1/500
