# Supplementary material for: Combined MEK and JAK/STAT3 pathway inhibition effectively decreases SHH medulloblastoma tumor progression
Source: Commun Biol. 2022 Jul 14;5:697. doi: 10.1038/s42003-022-03654-9 (PMC9283517; doi:10.1038/s42003-022-03654-9)
Supplement: Supplementary file 3 — Description of Additional Supplementary Files [file 42003_2022_3654_MOESM3_ESM.pdf]

Description of Additional Supplementary Files

File name: Supplementary Data 1-10
Description: All tables representing RNA seq and digital spatial profiling data depicted in figures.

File name: Supplementary Data 11
Description: All source data underlying the graphs and charts presented in main figures.
