# Supplementary material for: Combined MEK and JAK/STAT3 pathway inhibition effectively decreases SHH medulloblastoma tumor progression
Source: Commun Biol. 2022 Jul 14;5:697. doi: 10.1038/s42003-022-03654-9 (PMC9283517; doi:10.1038/s42003-022-03654-9)
Supplement: Supplementary file 6 — Reporting Summary [file 42003_2022_3654_MOESM6_ESM.pdf]

nature portfolio

Corresponding author(s): Dr. Tamra Werbowetski-Ogilvie

Last updated by author(s): May 20, 2022

Reporting Summary

Nature Portfolio wishes to improve the reproducibility of the work that we publish. This form provides structure for consistency and transparency in reporting. For further information on Nature Portfolio policies, see our Editorial Policies and the Editorial Policy Checklist.

Statistics

For all statistical analyses, confirm that the following items are present in the figure legend, table legend, main text, or Methods section.

n/a	Confirmed	
☐	☒	The exact sample size (*n*) for each experimental group/condition, given as a discrete number and unit of measurement
☐	☒	A statement on whether measurements were taken from distinct samples or whether the same sample was measured repeatedly
☐	☒	The statistical test(s) used AND whether they are one- or two-sided<br>*Only common tests should be described solely by name; describe more complex techniques in the Methods section.*
☒	☐	A description of all covariates tested
☐	☒	A description of any assumptions or corrections, such as tests of normality and adjustment for multiple comparisons
☐	☒	A full description of the statistical parameters including central tendency (e.g. means) or other basic estimates (e.g. regression coefficient) AND variation (e.g. standard deviation) or associated estimates of uncertainty (e.g. confidence intervals)
☐	☒	For null hypothesis testing, the test statistic (e.g. *F*, *t*, *r*) with confidence intervals, effect sizes, degrees of freedom and *P* value noted<br>*Give P values as exact values whenever suitable.*
☒	☐	For Bayesian analysis, information on the choice of priors and Markov chain Monte Carlo settings
☒	☐	For hierarchical and complex designs, identification of the appropriate level for tests and full reporting of outcomes
☒	☐	Estimates of effect sizes (e.g. Cohen's *d*, Pearson's *r*), indicating how they were calculated

Our web collection on statistics for biologists contains articles on many of the points above.

Software and code

Policy information about availability of computer code

Data collection	See details below
Data analysis	• HISAT2 v2.1.0 was used to map reads from xenograft RNA-seq experiments and XenofilteR v1.6 was used to deconvolute tumour (human)-origin reads from reads originating from the mouse host. Reads were assigned to transcripts from GENCODE release 23 using FeatureCounts vs 1.5.2.<br>• Salmon v1.4.0 was used to quantify reads from tumorsphere RNA-seq experiments against GENCODE release 35 transcripts. Quantification data was read into R using the tximport library v1.18.0.<br>• RNA-seq fold change analysis was carried out in R v3.6.1 (xenograft) or v4.0.5 (tumorsphere) using DESeq2 v1.24.0 (xenograft) or 1.30.1 (tumorsphere). Fold changes in tumorspheres were shrunk with v1.12.0 of the apeglm software.<br>• Gene set enrichment analysis was performed with GSEA v4.1.0 and v7.4 of the MSigDb database. Enrichment maps (EM) were created in Cytoscape v3.8.2 with v3.3.3 of the EnrichmentMap plugin. Gene sets were clustered within each EM using v1.3.1 of the ClusterMaker2 plugin, and preliminary cluster annotation was extracted with the AutoAnnotate plugin v1.3.4.

For manuscripts utilizing custom algorithms or software that are central to the research but not yet described in published literature, software must be made available to editors and reviewers. We strongly encourage code deposition in a community repository (e.g. GitHub). See the Nature Portfolio guidelines for submitting code & software for further information.

Data

Policy information about availability of data

All manuscripts must include a data availability statement. This statement should provide the following information, where applicable:

- Accession codes, unique identifiers, or web links for publicly available datasets
- A description of any restrictions on data availability
- For clinical datasets or third party data, please ensure that the statement adheres to our policy

GSE165960: RNA sequencing data

Field-specific reporting

Please select the one below that is the best fit for your research. If you are not sure, read the appropriate sections before making your selection.

☒ Life sciences ☐ Behavioural & social sciences ☐ Ecological, evolutionary & environmental sciences

For a reference copy of the document with all sections, see nature.com/documents/nr-reporting-summary-flat.pdf

Life sciences study design

All studies must disclose on these points even when the disclosure is negative.

Sample size	No power calculations were utilized to determine required sample size for in vitro and in vivo experiments. For in vivo studies, sample size was determined based on availability of animals and cost of the drug. For in vitro studies, all experiments were performed on a minimum of 3 independent biological replicates for each cell line with the majority of cell culture-based assays also being repeated with an additional 2 independent cell lines accordingly. For patient sample transcriptome and proteomics data, sample size was based on the number of samples already present in publicly available datasets or made available to the lab through collaboration.
Data exclusions	No exclusions.
Replication	For vitro/molecular experiments (ie. tumorsphere assays, bulk RNA sequencing), studies were carried out in 3 or greater independent biological replicates for each cell line with the majority also being replicated in 1-2 additional cell lines for reproducibility. All statistics were performed on independent biological replicates. Bulk RNA sequencing was performed on drug-treated tumorspheres in N=4 (vehicle, seluemtinib, pacritinib/selumetinib) or N=6 (pacritinib) biological replicates. In vivo studies evaluating the effect of the combination therapy on UI226 xenografts were performed 3 times with different endpoints due to COVID-imposed shutdowns, while the combination therapy study in the RCMB18 PDX model was performed once.
Randomization	Following intracerebellar transplantation, NOD SCID or NSG mice were randomly distributed into groups for drug treatments. For all other experiments, cells/samples were randomly assigned into groups.
Blinding	Blinding was performed during MRI imaging and tissue preparation for IHC. Remaining studies were not blinded but replicated as described above and/or repeated in 1-2 additional cell lines.

Reporting for specific materials, systems and methods

We require information from authors about some types of materials, experimental systems and methods used in many studies. Here, indicate whether each material, system or method listed is relevant to your study. If you are not sure if a list item applies to your research, read the appropriate section before selecting a response.

Materials & experimental systems

n/a	Involved in the study	
☐	☒	Antibodies
☐	☒	Eukaryotic cell lines
☒	☐	Palaeontology and archaeology
☐	☒	Animals and other organisms
☐	☒	Human research participants
☒	☐	Clinical data
☒	☐	Dual use research of concern

Methods

n/a	Involved in the study	
☒	☐	ChIP-seq
☒	☐	Flow cytometry
☐	☒	MRI-based neuroimaging

Antibodies

Antibodies used	The following antibodies were used in this study:

	Immunoblotting<br>STAT3 Cell Signaling Technology (CST) (4904S) 1/1000<br>p-STAT3 CST (4113S) 1/1000<br>pSTAT3 CST (9145S) 1/1000<br>pERK1/2 CST (4370) 1/1000<br>ERK 1/2 CST (4695)1/1000<br>Cleaved Caspase 3 CST (9664) 1/1000<br>GAPDH SCBT (sc-47724) 1/1000<br><br>Immunoblotting secondary antibodies<br>Goat anti-mouse HRP Abcam (ab6789) 1/3000<br>Donkey anti-rabbit HRP Jackson ImmunoResearch (711-035-152) 1/5000<br><br>Immunohistochemistry<br>STEM121 Clontech (Y40410) 1/500<br>Immunohistochemistry secondary antibodies<br>Biotin-SP sheep anti-mouse IgG Jackson ImmunoResearch (515-065-003) 1/500<br><br>Annexin V kit - BD Biosciences -561012 N/A, as per manufacturer's guidelines
Validation	p-STAT3 (CST 4113S and 9145), STAT3(CST 4904S) , pERK1/2 (CST 4370), ERK1/2 (CST 4695), Cleaved Caspase-3 (CST 9664) and GAPDH (SCBT sc-47724) have been widely used in publications.<br><br>To ensure product performance, all antibodies from CST are subject to the "Antibody Performance Guarantee" in which antibodies are validated in-house with multiple research applications.<br><br>The Stem121 (Y40410) antibody has been widely used for detection of human cell engraftment: Kelly S, et al . "Transplanted human fetal neural stem cells survive, migrate, and differentiate in ischemic rat cerebral cortex". PNAS . (2004) 101: 11839-11844.; 2. Cummings BJ, et al . "Human neural stem cells differentiate and promote locomotor recovery in spinal cord-injured mice". PNAS. (2005) 102: 14069-14074.; 3. Tamaki SJ, et al . "Neuroprotection of host cells by human central nervous system stem cells in a mouse model of infantile neuronal ceroid lipofuscinosis". Cell Stem Cell. (2009) 5: 310-319.; 4. Kallur T, et al . "Human fetal cortical and striatal neural stem cells generate region-specific neurons in vitro and differentiate extensively to neurons after intrastriatal transplantation in neonatal rats" .J Neurosci Res. (2006) 84:1630-1644. ; 5.SalazarDL,etal. "Human neural stem cells differentiate and promote locomotor recovery in an early chronic spinal cord injury NOD-scid mouse model" .PLoS ONE. (2010)5:e12272.<br><br>Annexin V (561012) was titrated by investigators to obtain appropriate concentrations

Eukaryotic cell lines

Policy information about cell lines

Cell line source(s)	HDMB03 was kindly provided by Dr. Till Milde (Milde T, et al., Journal of Neuro-oncology, 2012).<br>UI226 (SHH MB as analyzed by NanoString58) cells were a kind gift from Dr. Timothy Ryken (Dartmouth-Hitchcock Medical Center, New Hampshire, USA).<br>Daoy was purchased from the American Type Culture Collection (ATCC, Rockville, MD, USA).
Authentication	All cell lines have been authenticated by STR profiling (ATCC)
Mycoplasma contamination	Cell lines were not tested for Mycoplasma contamination
Commonly misidentified lines<br>(See ICLAC register)	No cell lines utilized in our study are listed in the ICLAC register

Animals and other organisms

Policy information about studies involving animals; ARRIVE guidelines recommended for reporting animal research

Laboratory animals	NOD-SCID male mice aged 7-9 weeks were utilized for all orthotopic xenografts involving UI226 and HDMB03 cells.<br>NSG male mice aged 7-9 weeks were utilized for in vivo orthotopic studies using the RCMB18 SHH MB PDX cell line.
Wild animals	Our study did not utilize wild animals
Field-collected samples	Our study did not involve field-collected samples
Ethics oversight	All in vivo procedures were approved by the University of Manitoba Animal Care Committee (AUP-20-023).

Note that full information on the approval of the study protocol must also be provided in the manuscript.

Human research participants

Policy information about studies involving human research participants

Population characteristics	These data are based on the samples collected in 2 recently published studies:<br>1. Cavalli et al., Cancer Cell 2017 Jun 12;31(6):737-754.e6. doi: 10.1016/j.ccell.2017.05.005<br>2. Petralia et al., Cell 2020 Dec 23;183(7):1962-1985.e31. doi: 10.1016/j.cell.2020.10.044. Epub 2020 Nov 25.
Recruitment	All medulloblastoma samples were collected at diagnosis after obtaining informed consent from subjects as part of the Medulloblastoma Advanced Genomics International Consortium. Approval was obtained from institutional research ethics boards at all contributing institutions (Cavalli et al, 2017).<br><br>For the Petralia et al., study, samples were obtained from the Children's Brain Tumor Network (CBTN) at the Children's Hospital of Philadelphia (CHOP). The patient selection was built based on specimen availability. In total, 226 samples from 204 pediatric subjects treated surgically and clinically at the Children's Hospital of Philadelphia were selected for analyses. Details are taken from Petralia et al., 2020.
Ethics oversight	See 2 published studies above for all details.

Note that full information on the approval of the study protocol must also be provided in the manuscript.

Magnetic resonance imaging

Experimental design

Design type	Resting state
Design specifications	*Specify the number of blocks, trials or experimental units per session and/or subject, and specify the length of each trial or block (if trials are blocked) and interval between trials.*
Behavioral performance measures	*State number and/or type of variables recorded (e.g. correct button press, response time) and what statistics were used to establish that the subjects were performing the task as expected (e.g. mean, range, and/or standard deviation across subjects).*

Acquisition

Imaging type(s)	Structural
Field strength	7 Tesla
Sequence & imaging parameters	Fast spin echo (FSE) T2, 256 x 256 matrix, 300um slice thickness, TE= 45, TR=5000, flip angle=90
Area of acquisition	Whole brain

Diffusion MRI ☐ Used ☒ Not used

Preprocessing

Preprocessing software	Data acquired using Preclinical Scan (MR Solutions), no processing applied.
Normalization	Not applicable
Normalization template	Not normalized
Noise and artifact removal	Not applicable
Volume censoring	Not applicable

Statistical modeling & inference

Model type and settings	Not applicable
Effect(s) tested	Not applicable

Specify type of analysis: ☐ Whole brain ☒ ROI-based ☐ Both

Anatomical location(s)	Tumor tissue visually identified and ROPI drawn using freehand tool in ImageJ. Tumor tissue, consisting of 1 or multiple lobes, in the cerebellum was visually identified on MRI images and used to delineate tumor size using the freehand tool. For each tumor sample, 18 serial sections (300 µm thickness) were obtained by MRI and volume calculated for each slice containing visible tumor tissue. The sum from all the slices containing tumor tissue was used to calculate an overall tumor volume.
Statistic type for inference (See Eklund et al. 2016)	Not applicable
Correction	Not applicable

Models & analysis

n/a	Involved in the study
☒	☐ Functional and/or effective connectivity
☒	☐ Graph analysis
☒	☐ Multivariate modeling or predictive analysis
